# Role of Gender and Physical Activity Level on Cardiovascular Risk Factors and Biomarkers of Oxidative Stress in the Elderly

**DOI:** 10.1155/2020/1315471

**Published:** 2020-06-19

**Authors:** Antoine Raberin, Philippe Connes, Jean-Claude Barthélémy, Pia Robert, Sébastien Celle, David Hupin, Camille Faes, Chantal Rytz, Frédéric Roche, Vincent Pialoux

**Affiliations:** ^1^Laboratoire Interuniversitaire de Biologie de la Motricité (LIBM) EA7424, Team « Vascular Biology and Red Blood Cell », Univ Lyon, Université Claude Bernard Lyon 1, Villeurbanne, France; ^2^Laboratoire d'Excellence du Globule Rouge (Labex GR-Ex), PRES Sorbonne, Paris, France; ^3^Institut Universitaire de France, Paris, France; ^4^Service de Physiologie Clinique et de l'Exercice, Faculté de Médecine Jacques Lisfranc, Univ Lyon, Université Jean Monnet, Saint-Etienne, France; ^5^Laboratoire Système Nerveux Autonome-Epidémiologie, Physiologie, Ingénierie, Santé, EA SNA-EPIS 4607, Université de Lyon, Université Jean Monnet Saint-Etienne, France; ^6^Department of Physiology and Pharmacology, Cumming School of Medicine, University of Calgary, Calgary, Alberta, Canada

## Abstract

**Background:**

Cardiovascular diseases remain as the leading cause of morbidity and mortality in industrialized countries. Ageing and gender strongly modulate the risk to develop cardiovascular diseases but very few studies have investigated the impact of gender on cardiovascular diseases in the elderly, which represents a growing population. The purpose of this study was to test the impact of gender and physical activity level on several biochemical and clinical markers of cardiovascular risk in elderly individuals.

**Methods:**

Elderly individuals (318 women (75.8 ± 1.2 years-old) and 227 men (75.8 ± 1.1 years-old)) were recruited. Physical activity was measured by a questionnaire. Metabolic syndrome was defined using the National Cholesterol Education Program Expert Panel's definition. Polysomnography and digital tonometry were used to detect obstructive sleep apnea and assess vascular reactivity, respectively. Blood was sampled to measure several oxidative stress markers and adhesion molecules.

**Results:**

The frequency of cardiovascular diseases was significantly higher in men (16.4%) than in women (6.1%) (*p* < 0.001). Body mass index (25.0 ± 4.3 vs. 25.8 ± 3.13 kg.m^−2^) and glycaemia (94.9 ± 16.5 vs. 101.5 ± 22.6 mg.dL^−1^) were lower, and High Density Lipoprotein (HDL) (74.6 ± 17.8 vs. 65.0 ± 17.2 mg.dL^−1^) was higher in women compared to men (*p* < 0.05). Oxidative stress was lower in women than in men (uric acid: 52.05 ± 13.78 vs. 59.84 ± 13.58, advanced oxidation protein products: 223 ± 94 vs. 246 ± 101 *μ*mol.L^−1^, malondialdehyde: 22.44 ± 6.81 vs. 23.88 ± 9.74 nmol.L^−1^). Physical activity was not associated with lower cardiovascular risk factors in both genders. Multivariate analyses showed an independent effect of gender on acid uric (*β* = 0.182; *p* = 0.020), advanced oxidation protein products (*β* = 0.257; *p* < 0.001), and HDL concentration (*β* = −0.182; *p* = 0.026).

**Conclusion:**

These findings suggest that biochemical cardiovascular risk factors are lower in women than men which could explain the lower cardiovascular disease proportion observed in women in the elderly.

## 1. Introduction

Cardiovascular diseases (CVD) remain the leading cause of morbidity and mortality in industrialized countries despite aggressive prevention strategies [1]. Several modifiable cardiovascular risk factors have been identified such as hypertension, diabetes, hyperlipidaemia, smoking, oxidative stress, obstructive sleep apnea (OSA), and sedentary lifestyle [2]. Atherosclerosis and endothelial dysfunction are the major pathophysiological mechanisms responsible for adverse cardiovascular events such as infarct or stroke. Atherosclerosis is a chronic, slowly progressive inflammatory disease that affects large and small blood vessels. It has been demonstrated that oxidative stress and cellular adhesion to the endothelium are involved in atherosclerosis and plaque progression [3] and represent biochemical cardiovascular risk factors.

Ageing and gender strongly modulate the risk to develop cardiovascular disease [2]. Arterial stiffness increases with ageing and causes endothelial cells to lose their defence capacity against oxidative stress [4]. Before menopause, women have a lower risk to develop cardiovascular disease than men because of the protective antioxidant effects of female hormones [5]. However, the decrease of female hormones occurring after menopause would increase the risks for cardiovascular diseases and death in this population [6]. In this context, although gender effects on ageing have been widely studied in middle-aged people, very few studies focused on the impact of gender on cardiovascular diseases in the elderly, particularly after 75 years old [7–9]. Cardiovascular diseases represent a major public health issue in a population of 75 years old [9].

Moreover, the amount of physical activity performed by any individual, as well as their respective physical fitness, decreases with ageing [10]. The World Health Organization recommends people of 65 years old and older to regularly practice any form of physical activity (leisure time physical activity, transportation, occupational, household chores, sports, etc.) to improve cardiorespiratory and muscle function and reduce the risks of chronic diseases [11]. However, the interaction of both gender and physical activity on cardiovascular diseases and risk factors in the elderly are poorly described. The purpose of this study was therefore to test the impact of gender and physical activity level on several biochemical and clinical markers of cardiovascular risk factors in elderly individuals. We hypothesized that women of 75 years old would exhibit enhanced cardiovascular risk factors compared to men of similar age, because of the loss of female hormone cardioprotective effect due to menopause.

## 2. Materials and Methods

### 2.1. Protocol

**Figure 1:**
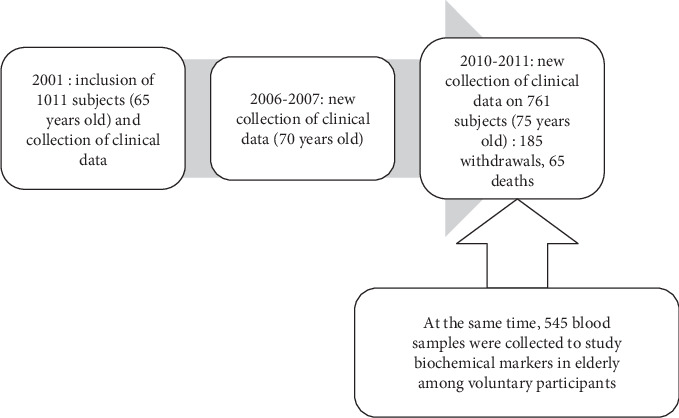
Study flowchart.

Our study was part of the PROgnostic indicator OF cardiovascular and cerebrovascular events (PROOF) study [12]. PROOF survey is a prospective longitudinal cohort study of 1,011 elderly subjects (mean age upon study inclusion in 2001: 65.64 ± 0.8). Subjects with previous cardiac events, stroke, type 1 diabetes, Parkinson's disease, or with life-expectancy of less than 5 years, and those who were dependent or living in a retirement home were excluded from the study. This study was approved by the Ethics Committee (CCPRB, Loire, France), and all subjects gave their written informed consent to participate. Volunteers were recruited in 2001 from the electoral list of the city of Saint-Etienne, France, and were eligible if aged 65 at the inclusion date. Inclusions were regular over a 2-year period, which was the time needed for the evaluation of the full cohort. A new collection of clinical complications and biomarkers was done in 2010-2011 on volunteers (mean age: 75.83 ± 1.22) at a hospital. At the same time, to evaluate the interaction between oxidative stress and cardiovascular risk factors, 545 subjects of the PROOF survey accepted to participate in the present study. For this purpose, blood samples were collected in 545 individuals from the right antecubital vein after a 12 h fasting: 318 women and 227 men (Figure 1). To obtain a large panel of cardiovascular risk factors and test the impact of gender, we evaluated biochemical (oxidative stress, circulating adhesion molecules, glycaemia, triglycerides, and cholesterol) and clinical parameters (treatment, metabolic syndrome components, overweight, blood pressure, physical activity practice, vascular function, and obstructive sleep apnea) in each individual.

### 2.2. Anthropometric and Clinical Examination

Height, weight, waist circumference, and body mass index (BMI; weight in kilograms divided by squared height in meters) are presented in Table 1. Waist circumference was measured midway between the iliac crest and the lowest rib, and the average of two measurements was used with an accuracy of 0.1 cm.

Medical histories, examinations, and treatments were recorded during each clinical visit to the research centre. Missing information was obtained from hospital charts, reviews, and from questionnaires sent to family practitioners. Diabetes, hypertension, thrombus, lipid, prostate, thyroid, anti-inflammatory, gout, bones disorder, pain sedative, depression treatment, and mineral supplement were used among our participants (Table 1). Participants were classified according to 4 categories by physicians: healthy, cardiovascular, cancer, or neurodegenerative.

Systolic blood pressure (SBP) and diastolic blood pressure (DBP) were measured in each individual. Arterial blood pressure was determined following the American Heart Association recommendation [13]. The presence of metabolic syndrome was highlighted when subjects met at least three of the criteria defined by the National Cholesterol Education Program Expert Panel (NCEP): high blood pressure (systolic ≥ 130 mmHg, diastolic ≥ 85 mmHg), triglycerides blood concentration > 150 mg.dL^−1^, fasting glycaemia ≥ 100 mg.dL^−1^, high waist circumference (men ≥ 102 cm, women ≥ 88 cm), and low High Density Lipoprotein (HDL) (men < 40 mg.dL^−1^, women < 50 mg.dL^−1^) [14]. A polysomnography was performed for each subject to assess the presence of obstructive sleep apnea (HypnoPTT device, Tyco Healthcare). Several indices were calculated: obstructive apnea-hypopnea index (oAHI), oxygen desaturation index (ODI), time spent at an oxygen saturation level <90%, mean saturation level, and minimum saturation level. Apnea was defined as a complete cessation of respiratory flow for ≥10 seconds. Hypopnea was defined as a decrease in amplitude of respiratory flow ≥50% during ≥10 sec associated with a 3% oxygen desaturation [15]. The desaturation index was defined as the number of desaturation events >3%/h. Results were communicated to the subject's primary care physician and treatment initiation was at their discretion.

### 2.3. Vascular Function

Vascular reactivity was measured by digital tonometry through a noninvasive method using the EndoPAT 2000 (EndoPAT, Itamar Medical, Atlanta, USA). This device measures peripheral arterial tonometry, which allows the calculation of reactive hyperaemia index (RHI, arbitrary units). The principle of peripheral arterial tonometry has already been described [16]. The system is composed of finger probes that assess digital volume changes occurring with pulse waves.

### 2.4. Physical Activity Evaluation

Physical activity (PA) was measured using the Population Physical Activity (POPAC) questionnaire and analysed with dedicated software. This questionnaire was validated [17] by the American College of Sports medicine guideline [18] and is based on the type of physical activity performed. It allows the calculation of five different components of physical activity over 7 days: daily energy expenditure and energy expenditure corresponding to low-intensity activities (low I PA) (<3 metabolic equivalents (METs)), moderate-intensity activities (mod I PA) (3 ≤ METs < 5), and vigorous-intensity activities (vig I PA) (≥5 METs). This questionnaire is designed to provide a complete picture of a subject's usual physical activity and also a moderate to vigorous physical activity (MVPA) measurement in MET-h/week.

### 2.5. Blood Lipids and Glucose

Standard enzymatic methods were used for serum total cholesterol, triglycerides (TGs), glucose concentration, and high-density lipoprotein cholesterol (HDL-C) with a Cobas Integra 400+® analyzer (Roche Diagnostics Gmbh, Mannheim, Germany). HDL-C was measured using the cholesterol enzymatic method after a selective immune-separation in homogenous phase. Low-density lipoprotein cholesterol (LDL-C) was calculated using the Friedewald formula ([LDL] = [Total Cholesterol] − [HDL] − ([TGs]/5)).

### 2.6. Adhesion Molecules, Oxidative Stress, and Nitric Oxide

The soluble forms of several adhesion molecules (soluble vascular cell adhesion molecule-1 (sVCAM-1), sP-selectin, soluble intercellular cell adhesion molecule-1 (sICAM-1), and sE-selectin) were measured by enzyme-linked immunosorbent assay using Eli-pair kits (Gen-probe Diaclone SAS, France) according to the manufacturer's instructions.

Advanced oxidation protein products (AOPP), malondialdehyde (MDA), ferric-reducing antioxidant power (FRAP), glutathione peroxidase (GPX), superoxide dismutase (SOD), uric acid (UA), and nitric oxide were assessed as previously described [19]. The plasma AOPP were determined by spectrophotometry and were calibrated with a chloramine-T solution that absorbs at 340 nm in the presence of potassium iodide. The absorbance of the reaction was read at 340 nm. AOPP concentrations were expressed as *μ*mol·L^−1^ of chloramine-T equivalents. The intra-assay coefficient of variation (CV) was 5.4%. Concentrations of plasma MDA, as thiobarbituric reactive substances, were determined by extracting the pink chromogen with n-butanol and measuring its absorbance at 532 nm by spectrophotometry using 1,1,3,3-tetraethoxypropan as standard. The intra-assay CV was 2.2%. FRAP plasma concentrations were measured at a controlled temperature (37°C) by spectrophotometry. FRAP concentrations were calculated using an aqueous solution of known Fe^2+^ concentration (FeSO_4_, 7H_2_O_2_) as standard at a wavelength of 593 nm. The intra-assay CV was 2.9%. Plasma GPX activity was determined as the rate of oxidation of NADPH to NADP+ after the addition of glutathione reductase (GR), reduced glutathione (GSH), and NADPH, using H_2_O_2_ as a substrate. The intra-assay CV was 4.6%. Plasma SOD activity was determined by the degree of inhibition of the reaction between superoxide radicals, produced by a hypoxanthine—xanthine oxidase system, and nitroblue tetrazolium. The intra-assay CV was 5.6%. The concentration of plasma UA was determined using a commercially available kit (Biolabo, Maizy, France). As a product of purine metabolism during reoxygenation, the UA concentration reflects reactive oxygen species (ROS) production via xanthine oxidase pathway activation. The intra-assay CV was 0.9%. Nitric oxide (NO) metabolism was quantified as the sum of nitrite and nitrate (NOx) concentrations. After nitrate reduction by nitrate reductase, the fluorimetric quantification of NOx was based upon the reaction of nitrite with 2,3-diaminonaphthalene and sulfanilamide. The intra-assay CV was 5.4%.

### 2.7. Statistical Analysis

Distribution of medical status (healthy, cardiovascular, cancer, neurodegenerative) between men and women was analysed by the contingency table and chi^2^ test. Quantitative parameters were compared between men and women using Student's *t*-test or Wilcoxon's signed-rank test when data were non-normally distributed according to the Shapiro-Wilk test. Multivariate linear regression models were used to identify the independent effect of gender, clinical markers, and biomarkers on cardiovascular risk factors which differ between men and women. The risk of collinearity effect between variables was assessed by the calculation of the variance inflation factor (VIF). Each time two covariates showed a VIF > 2.5, one of them was not included in the model. For each dependent variable tested in linear regression models (RHI, AOPP, UA, MDA, or HDL), clinical markers (BMI, metabolic syndrome, MVPA, Low I PA, and oAHI), treatments (those who significantly impacted the dependent variable), and gender were considered as covariates. All the covariates showed a VIF < 1.5 in our models. A *p* value < 0.05 was considered statistically significant. Statistical analyses were performed using SPSS-version 17.0 for Windows (SPSS Inc., Chicago, IL, USA).

## 3. Results

### 3.1. Gender Effect

Height and weight were different between men and women (Table 1). The most frequent treatment in males and females are listed in Table 1. Thyroid therapy (*p* < 0.001), sedative (*p* = 0.039), bones disorder treatment (*p* < 0.001), and mineral supplements (*p* = 0.024) were more frequent in females than in males. In contrast, there was a greater proportion of men using antithrombotic molecules (*p* = 0.002), or undergoing gout treatment (*p* < 0.001) and prostate therapy (*p* < 0.001). No significant differences between males and females were observed for the other treatments listed.

The repartition of men and women in each disease category was different (*X*^2^ (3) = 20.9, *p* < 0.001, Table 2). Apart from being the most frequent disease category in the cohort (10.2% CVD, 5.8% Cancer, 1.3% Neurodegenerative), the frequency of CVD was significantly higher in men than in women (*p* < 0.001).

BMI (*p* = 0.023) and resting glycaemia (*p* < 0.001) were both lower in women than in men. In addition, women had significantly higher LDL (*p* < 0.001) and HDL (*p* < 0.001) than men (Table 3). No difference was observed for SBP, DBP, or triglycerides.

Hypertension status was not significantly different between men and women (Table 4, *X*^2^ (1) = 2.4, *p* = 0.122). In contrast, the proportion of those with metabolic syndrome was higher in men than in women (*X*^2^ (1) = 3.9, *p* < 0.05). oAHI (*p* < 0.001) and ODI (*p* < 0.001) were significantly lower, and RHI (*p* < 0.01) was significantly higher in women than in men (Table 4).

While plasma adhesion molecules concentrations did not differ between men and women (Table 5), women had lower plasma concentrations of UA (*p* < 0.001), MDA (*p* = 0.046), AOPP (*p* = 0.008), and FRAP (*p* < 0.001) than men (Table 5). No difference was observed between men and women for GPX, SOD, and NO.

Women practiced more physical activity (PA) of low intensity (*p* = 0.020) than men (Table 3). However, they practiced less moderate (*p* < 0.001) and vigorous (*p* < 0.001) PA than men. This result was confirmed by the higher moderate to vigorous PA (MVPA) (*p* < 0.001) found in men.

### 3.2. Multivariate Linear Regression Models

Several multivariate linear regression models were performed between clinical and biological parameters (Table 6).

The first model tested included RHI as the dependent variable and contained gender, BMI, metabolic syndrome, MVPA, Low I PA, and oAHI as covariates. The overall model was not statistically significant (*R*^2^ = 0.067; *p* = 0.125). A second model was tested to identify the independent predictors of AOPP. This model included gender, BMI, metabolic syndrome, MVPA, Low I PA, oAHI, and diabetes treatment as covariates. The overall model was statistically significant (*R*^2^ = 0.370; *p* < 0.001), and gender (*β* = 0.182; *p* = 0.020; *β* IC 95% [0.03; 0.33]), metabolic syndrome (*β* = 0.513; *p* < 0.001; *β* IC 95% [0.35; 0.66]), and diabetes treatment (*β* = −0.219; *p* = 0.004; *β* IC 95% [-0.36; -0.07]) remained independently associated with AOPP. A third model focusing on the determinants of UA included gender, BMI, metabolic syndrome, MVPA, Low I PA, oAHI, hypertension treatment, thrombus treatment, bones disorders treatment, and mineral supplement as covariates. The overall model was statistically significant (*R*^2^ = 0.234; *p* < 0.001) with gender (*β* = 0.257; *p* < 0.001; *β* IC 95% [0.11; 0.45]) and hypertension treatment (*β* = 0.355; *p* < 0.001; *β* IC 95% [0.17; 0.49]) being independently associated with UA. MDA was included as the dependent variable in a fourth model with gender, BMI, metabolic syndrome, MVPA, Low I PA, and oAHI as covariates. The overall model was not statistically significant (*R*^2^ = 0.076; *p* = 0.073). Finally, a fifth model including HDL as the dependent variable was tested and contained gender, BMI, MVPA, Low I PA, oAHI, gout treatment, hypertension treatment, bones disorders treatment, and mineral supplement as covariates. The overall model was statistically significant (*R*^2^ = 0.217; *p* < 0.001), and gender (*β* = −0.142; *p* = 0.026; *β* IC 95% [-0.32; -0.03]), BMI (*β* = −0.206; *p* < 0.001; *β* IC 95% [-0.31; -0.02]), MVPA (*β* = 0.133; *p* = 0.044; *β* IC 95% [0.003; 0.29]), gout treatment (*β* = −0.193; *p* = 0.030; *β* IC 95% [-0.28; -0.01]), and bone disorder treatment (*β* = 0.141; *p* = 0.035; *β* IC 95% [0.04; 0.29]) remained independently associated with HDL.

## 4. Discussion

There are few studies conducted on European cohorts and focusing on the associations between oxidative stress, vascular adhesion molecules, and clinical markers, and even less on population over 75 years old [7, 20, 21]. Our study demonstrated a lower frequency of cardiovascular disease in women than in men at 75 years old. This result might be explained by an independent effect of gender on HDL (higher in females) and oxidative stress (lower in females) levels. This gender effect was also observed in the healthy part of the cohort (data not shown) which highlighted its independence regarding cardiovascular diseases. Women also practiced more PA at low intensity and less moderate to vigorous PA than men. However, none of the clinical/biological risk factors studied in multivariate analyses were independently affected by low-intensity PA, and only HDL was independently affected by moderate to vigorous PA.

The incidence of cardiovascular disease has previously been reported to be higher in middle-aged men than in middle-aged women [22], yet no information is available in elderly people. This difference seen in the middle-aged population could be explained by several factors including (1) detrimental behavioural differences between men and women with lower alcohol and tobacco consumption and lower time at work for women, even though these differences tend to reduce between both genders [23]; (2) sex chromosome involvement in CVD incidence independent of their effect on sexual hormones [24]: for example, the Y chromosome has been shown to be involved in the development of hypertension; (3) oestrogen levels which can modulate the risk to develop CVD. Moreover, a Danish cohort study reported that postmenopausal women had less infarction, heart failure, or death risk after hormonal therapy compared to before hormonal therapy [25]. This risk reduction could be explained by the antioxidant power of female hormones [5]. However, hormone replacement therapy remained a controversial topic and could be harmful in postmenopausal women [26]. Although middle-aged men would be prone to a higher risk of CVD development than middle-aged women [22], this difference has been suggested to be reduced after menopause [27]. The loss of female hormone antioxidant power after menopause likely increases cardiovascular risk in women [27]. Literature advanced that after 45 years for men and 55 years for women, the risk for CVD increases similarly in both, men and women [28]. However, very few studies focused on the elderly population aged over 75 years, and it is unknown whether CVD prevalence is also affected by gender. An American cohort study showed that CVD prevalence was the same between men and women aged 60-79 years old [9]. In contrast, our results drawn from a French population demonstrated that old women (75 years old) are more protected from CVD than old men, as it is the case in the middle-aged population [9].

Our study showed that women aged 75 years old were less affected by metabolic syndrome than men, while other studies in Taiwanese and Chinese [29, 30] cohorts reported that women over 65 years old were more prone to metabolic syndrome. Although the difference in age between these studies could explain these discrepancies, one may note that the genetic background and lifestyle are very different between Asian and Caucasian populations. In contrast, and in agreement with our findings, another French cohort study showed a significant difference in the prevalence of metabolic syndrome between men (14.1%) and women (12%) older than 70 years old [31]. In addition, the higher blood HDL levels found in women could have played a cardioprotective effect in this population due to its antioxidant characteristics [32], thereby lowering the frequency of cardiovascular disease. Indeed, HDL is known to negatively correlate to CVD in both men and women [33], moreover, our results were supported by the stronger relationship in women and in all ages including the elderly.

In agreement with the literature in adults [34], and despite the fact that OSA prevalence increases after menopause in women, our data confirmed that OSA is more frequent in males than in females. OSA strongly modulates oxidative stress [35], and we suspected that differences in OSA frequency between women and men could also explain the differences in oxidative stress observed between the two groups. However, multivariate models failed to show an independent association between OSA and oxidative stress markers.

Women were also characterized by higher vascular reactivity than men, which could explain why, in our cohort, the frequency of cardiovascular disease was lower in women than in men. Although the vascular system seems to be better preserved in females than in males, we did not find any difference in the level of nitric oxide end-products and cellular/vascular adhesion molecules between the two groups. This is quite surprising since impaired vascular function or lower vascular activity is usually accompanied by increased circulating levels on several proadhesive molecules [36]. The reasons of the lower vascular reactivity found in men are not identified in this study since none of the covariates included in the multivariate model were independently associated with vascular reactivity. Yoshino et al. reported sex-specific genetic variants associated with coronary endothelial dysfunction [37] and Wang et al. found sex-specific differences in microvascular endothelial cell phosphodiesterase due to gonadal hormone [38]. Thus, one could hypothesize that sex-specific genetic variants could participate in the vascular reactivity differences observed between women and men in our study.

Finally, we observed that women practiced less moderate and vigorous-intensity PA than men. Moderate and vigorous-intensity PA is recognized as a beneficial modulator of cardiovascular health [39]. However, we also found that women practiced more PA at low intensity compared to men. Several studies reported beneficial health consequences of low-intensity PA [40, 41]. We found no independent effect of PA level in any of the biological/clinical markers measured in this study. This result could be explained by the fact that both women and men practiced more PA than the guidelines set for the healthy population (respectively, 3.536 h/week, 7.085 h/week vs. 2.5 h/week), and it is thus difficult to observe an impact of PA with such level of practice.

This study reported unexpected results. In particular, women exhibited lower cardiovascular risk factors than men, and we did not find any impact of physical activity on biochemical risk factors excepted for HDL. Although a complete package of cardiovascular risk factors was explored including metabolic syndrome component, adhesion molecule, oxidative stress, obstructive sleep apnea, and physical activity, several limitations deserve further discussion. A part of our population followed some treatment: mainly for hypertension (25.7%), dyslipidaemia (15.0%), and thrombus (10.4%). It should be acknowledged that these treatments could affect oxidative stress markers although they have been included as covariates in the multivariate linear regression models when necessary. PA was evaluated by a questionnaire in the present study, and a previous work showed that this method can overestimate or underestimate PA intensity in men and women, respectively [42]. A recent meta-analysis also confirmed that objective measures like heart rate, indirect calorimetry, or accelerometry in the elderly may be more accurate to describe their PA practice [43]. In addition, the recruitment of our study was based on voluntary participation. In consequence, our population may have a higher socioeconomic status than the general population of this age group and may not fully reflect the French population of 75 years old. Furthermore, longitudinal analysis should be used in a future study to follow cardiovascular risk evolution with ageing.

## 5. Conclusion

Our results showed for the first time an impact of gender on cardiovascular risk factors in the elderly. Gender impact led to the difference in biochemical cardiovascular risk factors between men and women. Thus, the lower cardiovascular disease proportion observed in women than in men could be attributed to the higher HDL levels, lower oxidative stress, and lower OSA and metabolic syndrome frequency found in women.

## Figures and Tables

**Table 1:** Anthropometric characteristic and treatment frequency in men and women.

	Women	Men	*p* value	*N*
Age (yr)	75.8 ± 1.2	75.8 ± 1.1	0.637	545
Height (cm)	159 ± 6	170 ± 6	<0.001	545
Weight (kg)	63.6 ± 11	74.9 ± 1	<0.001	545
Waist circumference (cm)	84.7 ± 10	90.6 ± 9	<0.001	545
Diabetes treatment	3.3%	3.2%	0.560	545
Thrombus treatment	7.9%	14.2%	0.002	545
Hypertension treatment	23.5%	28.9%	0.076	545
Lipid treatment	13.8%	16.7%	0.208	545
Prostate treatment	—	7.2%	<0.001	545
Thyroid treatment	7.2%	1.7%	<0.001	545
Anti-inflammatory treatment	7.2%	4.5%	0.075	545
Gout treatment	0.7%	5%	<0.001	545
Bone disorder treatment	5.4%	1%	<0.001	545
Pain treatment	5.4%	4.2%	0.395	545
Sedative treatment	6.3%	3.2%	0.039	545
Depression treatment	5.3%	2.7%	0.087	545
Mineral supplement	6.9%	3.5%	0.024	545

Age, high, weight, and waist circumference are presented as mean ± SD. Treatments represent the percentage of subject with medication in the corresponding group.

**Table 2:** Repartition of gender in the different disease families and healthy state.

	Healthy	Cancer	Cardiovascular	Neurodegenerative
Women	86.9%	5.6%	6.1%	1.4%
Men	76.3%	6%	16.4%	1.3%

*X* ^2^ (3) = 20.9, *p* < 0.001.

**Table 3:** BMI, arterial blood pressure, biochemical parameters, and physical activity data in women and men.

	Women	Men	*p* value	*N*
BMI (kg.m^−2^)	24.6 (22.0-27.3)	25.6 (23.7-27.8)	0.023	545
SBP (mmHg)	135.18 ± 14.22	133.71 ± 14.375	0.361	545
DBP (mmHg)	80.0 (76.0-84.0)	81.0 (78.0-84.2)	0.113	545
Triglycerides (mg.dL^−1^)	93 (75-123)	95 (69-132)	0.619	545
HDL (mg.dL^−1^)	74.6 ± 17.8	65.0 ± 17.2	<0.001	545
LDL (mg.dL^−1^)	138.5 ± 33.9	124.9 ± 31.3	<0.001	545
Glycaemia (mg.dL^−1^)	91 (86-99)	97 (90-105)	<0.001	545
Low I PA (h.day^−1^)	5.276 ± 1.82	4.994 ± 1.64	0.020	545
Mod I PA (h.week^−1^)	2.0 (0.2-4.0)	3.0 (1.0-7.7)	<0.001	545
Vig I PA (h.week^−1^)	0.0 (0.0-0.0)	0.0 (0.0-1.5)	<0.001	545
MVPA (MET-h.week^−1^)	10.5 (3.7-23.5)	22.2 (9.2-53.2)	<0.001	545

Data are presented as either mean ± SD or median (Quartile 1–Quartile 3). BMI: body mass index; DBP: diastolic blood pressure; HDL: high-density lipoprotein cholesterol; LDL: low-density lipoprotein cholesterol; Low I PA: low-intensity physical activity (<3 METs); Mod I PA: moderate-intensity physical activity (3 ≤ METs < 5); MVPA: moderate to vigorous physical activity; SBP: systolic blood pressure; Vig I PA: vigorous-intensity physical activity (≥5 METs); mod to vig PA.

**Table 4:** Obstructive syndrome apnea components and vasoreactivity data in women and men and frequency and percentage of hypertension and metabolic syndrome.

	Women	Men	*p* value	*N*
Hypertensive	33.2%	38.1%	0.122	545
Metabolic syndrome	7.3%	14.3%	0.046	545
oAHI nb events.h^−1^	10.5 (5.9-18.8)	17.9 (9.0-27.0)	<0.001	295
ODI nb events.h^−1^	6.6 (2.7-10.9)	9.0 (3.9-16.9)	0.001	295
RHI	1.870 ± 0.589	1.731 ± 0.437	0.010	369

RHI, ODI nb events, and oAHI nb events are presented as either mean ± SD or median (Quartile 1–Quartile 3). Hypertensive and metabolic syndromes represent the percentage of subject with these pathologies in the corresponding group. oAHI nb events.h^−1^, obstructive apnea-hypopnea index in one hour; ODI nb events.h^−1^, obstructive desaturation index in one hour; RHI, reactive hyperaemia index.

**Table 5:** Blood circulating adhesion molecules and oxidative stress markers in men and women.

	Women	Men	*p* value	*N*
sICAM-1 (ng.L^−1^)	1292.1 ± 513.0	1328.3 ± 522.2	0.428	529
sVCAM-1 (ng.L^−1^)	1100 (983-1175)	1115 (1005-1192)	0.078	322
sE-selectin (ng.L^−1^)	83.5 (34.5-115.5)	72.4 (30.4-116.0)	0.303	427
sP-selectin (ng.L^−1^)	40.4 ± 8.2	40.4 ± 8.5	0.628	322
UA (mg.L^−1^)	52.04 ± 13.78	59.84 ± 13.58	<0.001	545
MDA (nmol.L^−1^)	21.9 (18.3-26.2)	23.1 (19.0-27.1)	0.046	531
AOPP (*μ*mol.L^−1^)	198.1 (166.7-259.6)	219.9 (171.1-293.3)	0.008	528
FRAP (mmol.L^−1^)	684.10 ± 142.21	784.95 ± 144.36	<0.001	455
GPX (*μ*mol.mL^−1^.min^−1^)	8.893 ± 3.639	9.120 ± 4.136	0.506	529
SOD (*μ*mol^−1^.mL^−1^.min^−1^)	0.131 (0.112-0.147)	0.125 (0.107-0.147)	0.155	517
NOx (*μ*mol.L^−1^)	4.282 ± 1.432	4.065 ± 1.529	0.092	539

Data are presented as mean ± SD or median (Quartile 1–Quartile 3). AOPP: advanced oxidation protein product; FRAP: ferric reducing antioxidant power; GPX: glutathione peroxidase; MDA: malondialdehyde; NOx: nitric oxide metabolites; sICAM: soluble intercellular cell adhesion molecule-1; SOD: Superoxide dismutase; sVCAM: soluble vascular cell adhesion molecule-1; UA: uric acid.

**Table 6:** Multivariate linear regression models.

Dependent variable	Covariates	*R* ^2^	*p* value	Covariates bêta	Covariates *p* value
RHI	Gender, metabolic syndrome, BMI, MVPA, low I PA, oAHI	0.067	0.125	—	—

AOPP		0.370	<0.001	—	—
	Gender			0.182	0.020
	Metabolic syndrome			0.513	<0.001
	Diabetes treatment			-0.219	0.004
	BMI, MVPA, low I PA, oAHI			—	NS

UA		0.234	<0.001	—	—
	Gender			0.257	<0.001
	Hypertension treatment			0.355	<0.001
	Metabolic syndrome, BMI, MVPA, low I PA, gout treatment, bones disorder treatment, Thrombus treatment			—	NS

MDA	Gender, metabolic syndrome, BMI, MVPA, low I PA	0.076	0.073	—	—

HDL		0.217	<0.001		
	Gender			-0.142	0.026
	BMI			-0.206	<0.001
	MVPA			0.133	0.044
	Gout treatment			-0.193	0.030
	Bones disorder treatment			0.141	0.035
	Low I PA, hypertension treatment, oAHI			—	NS

AOPP: advanced oxidation protein product; BMI: body mass index; HDL: high-density lipoprotein cholesterol; Low I PA: low-intensity physical activity (<3 METs); MDA: malondialdehyde; MVPA: moderate to vigorous physical activity; RHI: reactive hyperaemia index; UA: uric acid. NS: *p* value > 0.05.

## Data Availability

The data used to support the findings of this study are available from the corresponding author upon request.
